# Adiposity, Body Composition Measures, and Breast Cancer Risk in Korean Premenopausal Women

**DOI:** 10.1001/jamanetworkopen.2024.5423

**Published:** 2024-04-05

**Authors:** Thi Xuan Mai Tran, Yoosoo Chang, Hye Rin Choi, Ria Kwon, Ga-Young Lim, Eun Young Kim, Seungho Ryu, Boyoung Park

**Affiliations:** 1 Department of Preventive Medicine, Hanyang University College of Medicine, Seoul, Republic of Korea; 2 Center for Cohort Studies, Kangbuk Samsung Hospital, Sungkyunkwan University School of Medicine, Seoul, Republic of Korea; 3 Department of Occupational and Environmental Medicine, Kangbuk Samsung Hospital, Sungkyunkwan University School of Medicine, Seoul, Republic of Korea; 4 Department of Clinical Research Design and Evaluation, Samsung Advanced Institute for Health Sciences and Technology, Sungkyunkwan University, Seoul, Republic of Korea; 5 Institute of Medical Research, Sungkyunkwan University, School of Medicine, Suwon, Republic of Korea; 6 Department of Surgery, Kangbuk Samsung Hospital, Sungkyunkwan University School of Medicine, Seoul, Republic of Korea; 7 Hanyang Institute of Bioscience and Biotechnology, Hanyang University, Seoul, Republic of Korea

## Abstract

**Objective:**

To investigate the association between body composition parameters and breast cancer (BC) risk in premenopausal women.

**Design, Setting, and Participants:**

Prospective cohort study using data from the Kangbuk Samsung Cohort Study. Participants were women aged 20 to 54 years who were enrolled from 2011 to 2019 and followed up for BC development until December 31, 2020. Data were analyzed from June to August 2023.

**Exposures:**

Trained nurses conducted anthropometric measurements and assessed body composition using segmental bioelectric impedance analysis. The analysis encompassed adiposity measures such as body mass index (BMI), waist circumference, and body composition parameters, including muscle mass, fat mass, ratio of muscle mass to weight, ratio of fat mass to weight, and fat mass index.

**Main outcomes and measures:**

Adjusted hazard ratios (aHR) for BC during the follow-up period.

**Results:**

Among 125 188 premenopausal women, the mean (SD) age was 34.9 (6.3) years. During a mean (range) follow-up of 6.7 (0.5-9.9) years, 1110 incident BC cases were identified. The mean (SD) BMI and waist circumference were 21.6 (3.1) and 75.3 (8.2) cm, respectively. Higher BMI and waist circumference were associated with decreased risk, with an aHR of 0.89 (95% CI, 0.84-0.95) per SD increase in BMI and 0.92 (95% CI, 0.86-0.98) per SD increase in waist circumference. A higher ratio of fat mass to weight was associated with decreased BC risk (aHR, 0.92; 95% CI, 0.86-0.99 per SD increase), whereas the opposite trend was observed for the ratio of muscle mass to weight, with an aHR of 1.08 (95% CI, 1.02-1.15) per SD increase. The results remained consistent even after additional adjustments for height in the model. The fat mass index was also inversely associated with BC risk, with an HR of 0.90 (95% CI, 0.85-0.97) per SD increase.

**Conclusions and Relevance:**

In this cohort study of premenopausal women, a higher level of adiposity, represented by increased BMI, waist circumference, and fat mass, was consistently associated with decreased breast cancer risk. Conversely, muscle mass and its ratio to weight displayed opposite or inconsistent patterns. These findings suggest an inverse association between excess adiposity and the risk of BC in premenopausal women, confirming earlier findings that BMI is an indirect measure of adiposity.

## Key Points

**Question:**

Are body composition parameters associated with breast cancer (BC) risk in premenopausal women?

**Findings:**

In this cohort study of 125 188 premenopausal women, a higher level of adiposity, represented by increased body mass index (BMI), waist circumference, and fat mass, was consistently associated with decreased BC risk. Conversely, muscle mass and its ratio to weight displayed opposite or inconsistent patterns.

**Meaning:**

These findings suggest an inverse association between excess adiposity and the risk of BC in premenopausal women, confirming earlier findings that BMI is an indirect measure of adiposity.

## Teaser

This cohort study evaluates the association between body composition parameters and breast cancer risk in premenopausal Korean women.

## Introduction

Obesity, commonly measured by body mass index (BMI), demonstrates contrasting associations with breast cancer risk depending on menopausal status.^1,2,3^ Accumulating evidence consistently indicates a positive association between increased adiposity and postmenopausal breast cancer.^1,2^ However, in premenopausal women, a higher BMI has been reported to be unrelated to breast cancer or associated with reduced breast cancer risk.^2,3^ Similar trends have been observed for waist circumference, an indirect measure of visceral adiposity.^4,5,6,7,8^ A positive association between waist circumference and breast cancer risk has been consistently noted in postmenopausal women; however, in premenopausal women, the association between waist circumference and breast cancer varies with or without adjustment for BMI.^4,5,6,7,8^

While BMI is widely used as a practical measure of obesity, it lacks the ability to differentiate between adiposity and muscle mass or provide information about fat distribution, making it an imperfect indicator for characterizing body composition and body fat distribution. It is crucial to highlight that premenopausal women typically have a higher proportion of lower body fat, often characterized by gluteofemoral fat, in contrast to visceral adiposity.^9^ Recent evidence suggests different associations between excess body fat and breast cancer in women according to their menopausal status. Studies have found that body fat levels are associated with an elevated risk of breast cancer in postmenopausal women,^10,11,12^ whereas no association was observed between body fat measurements and breast cancer before menopause.^11^ To date, the association between body composition measures and breast cancer risk remains unclear, with limited evidence for premenopausal women.

To our knowledge, the association between body composition measures and breast cancer risk in premenopausal women has not been evaluated, especially in Asian populations. This is important because Asian people typically have a higher total body fat^13^ and an elevated risk of cardiometabolic diseases^14^ compared with White individuals with the same BMI. Therefore, we examined the association between breast cancer risk and adiposity measures encompassing conventional metrics (BMI and waist circumference) and body composition measures assessed through segmental bioelectric impedance analysis in a prospective cohort of premenopausal Korean women.

## Methods

### Study Design and Population

This prospective cohort study involved a subsample from the Kangbuk Samsung Cohort Study, which was initiated in 2011 and has continuously recruited Korean men and women aged 18 years or older who underwent annual or biennial health examinations and provided informed consent for linkage to national databases for research purposes.^15,16^ As of 2023, the cohort has increased to approximately 360 000 participants. The majority of participants comprise employees of various companies across countries and their spouses, who were provided free annual or biennial health screening examinations under the Industrial Safety and Health Law in South Korea. This study was approved by the Institutional Ethics Committee of Kangbuk Samsung Hospital, which waived the requirement for informed consent specific to the current study because of the use of preexisting and deidentified data that were routinely collected during the health screening process and linked to the national cancer registry in Korea. This article was prepared following the Strengthening the Reporting of Observational Studies in Epidemiology (STROBE) reporting guideline for cohort studies.

**Figure 1.**
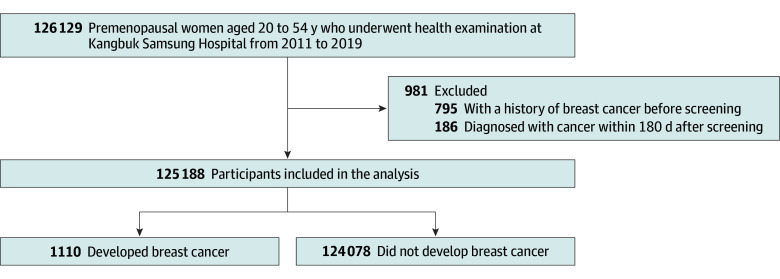
Diagram of the Study Population Selection

The initial dataset included 126 169 premenopausal women aged 20 to 54 years who underwent health examinations at Kangbuk Samsung Hospital between 2011 and 2019. Menopause was defined as a period of 12 months or more without menstruation, as reported through a self-administered structured questionnaire, where premenopausal status was defined as the presence of at least 1 menstrual cycle within the past 12 months. Women with a history of cancer before the screening were excluded from the analysis. In addition, we excluded breast cancer cases diagnosed within 180 days after screening to eliminate prevalent cancer cases at recruitment from the analysis. The final dataset included 125 188 women (Figure 1).

### Measures of BMI and Body Compositions

BMI and body composition were assessed at the first baseline health examination (date of cohort entry). Trained nurses measured height, weight, and body composition with participants wearing lightweight hospital gowns and no shoes. Height was measured to the nearest 0.1 cm using a stadiometer while participants stood barefoot. Waist circumference was measured to the nearest 0.1 cm at the midpoint between the bottom of the rib cage and above the top of the iliac crest, while the participants stood evenly on both feet, arms at their sides, and facing forward. Weight and body composition were assessed using a multifrequency bioelectrical impedance analysis (BIA) device (InBody 720; Biospace Inc) after all participants had fasted for at least 10 hours before measurement. The BIA device automatically calculates fat, muscle, and fat-free mass. BIA has been validated for body composition assessment, showing strong correlations with dual energy radiograph absorptiometry (DEXA) and abdominal CT measurements, including fat and muscle mass.^17,18^ In a previous study of 200 Korean adults aged 20 to 69 years, high correlation coefficients (*r* = 0.951 and *r* = 0.889 for men and *r* = 0.956 and *r* = 0.898 for women) demonstrated the validity of the lean body mass and percentage body fat measurements assessed using BIA and DEXA.^19^ The following body composition measures were included in the analysis: muscle mass and fat mass measured in kilograms, ratios of muscle mass to weight, and fat mass to weight. We further included fat mass index (FMI), calculated as fat mass in kilograms divided by height in meters squared.^20^

### Ascertainment of Incident Breast Cancer

Breast cancer case ascertainment was achieved through linkage to the Korea Central Cancer Registry (KCCR) by the resident registration number with data up to December 31, 2020. Since 1999, the KCCR has collected annual data on patients newly diagnosed with cancer from hospitals and regional cancer registries nationwide, with a coverage rate exceeding 98%.^21^ Incident breast cancer was defined according to the *International Statistical Classification of Diseases and Related Health Problems, Tenth Revision* codes for invasive breast cancer (C50) and ductal carcinoma in situ (D05.1).

In our cohort, the initial baseline visit was designated as cohort entry and the start date of the follow-up period. Following data linkage, the baseline visit dates were cross-referenced with breast cancer diagnoses obtained from the KCCR to identify individuals with new cases. Participants with a recorded cancer diagnosis preceding the baseline visit date were considered to have a history of breast cancer and were excluded from the initial analysis. Additionally, breast cancer cases documented within the first 6 months after the baseline visit were considered prevalent and excluded from the analysis. Incident cases refer to individuals who developed breast cancer more than 6 months after cohort entry. We calculated the person-years from the date of cohort entry (baseline health examination) to the date of breast cancer registration or the last date of follow-up (December 31, 2020).

### Measure of Covariates

Information on other covariates, including demographic characteristics and reproductive, family, and medical histories, was collected using standardized questionnaires during health examinations. The following factors were considered as covariates: age at the time of health examination, family history of breast cancer among first-degree relatives, number of children delivered, smoking status, alcohol consumption, physical activity, and age at menarche. Smoking status included never smokers, former smokers, or current smokers. The frequency of alcohol consumption per week was categorized as never, once or twice per week, and more than twice per week. Physical activity was measured using the validated Korean version of the International Physical Activity Questionnaire Short Form and categorized into 4 groups: inactive, minimally active, health-enhancing physically active, and missing.

### Statistical Analysis

Means and frequencies were used to summarize the study cohort characteristics in the total population and breast cancer development status. BMI, waist circumference, and body composition measures were analyzed as categorical (4 quartiles) and continuous variables. Because there is no uniformly accepted definition for a significant difference in waist circumference^22^ and body composition,^23^ a tolerance limit of a unit increase (10% increase for muscle mass divided by weight and fat mass divided by weight) and an SD increase were used for all studied measures. The breast cancer incidence rate was calculated for each quartile per 100 000 person-years. Age- and multivariable-adjusted hazard ratios (HRs) and 95% CIs for associations between anthropometries, body composition measures, and breast cancer risk were estimated using Cox proportional hazard regression. The underlying time scale was the time from the body composition measurement (date of health examination) to the time of breast cancer diagnosis or the last date of follow-up (December 31, 2020). Regression model 1 adjusted for age at enrollment. Model 2 was adjusted for age at enrollment, family history of breast cancer among first-degree relatives, number of children delivered, smoking status, alcohol consumption, physical activity, and age at menarche. For variables with missing data, we included missing-value indicators. We tested for linear trends across body composition quartile variables by entering a single ordinal term into the Cox regression model 2. As height is associated with an increased breast cancer risk,^24^ there are concerns that height might confound the association between body composition measures and breast cancer and should be considered in the analysis.^25,26^ To address this concern, we used another regression model (model 3) that was additionally adjusted for height (measured in centimeters). The proportional hazard assumption of the primary end point was evaluated by generating visual diagnostics such as log-log plots and plots of time-dependent regression coefficients for each variable in the model. No evidence of a violation of the proportional hazards assumption was reported. Statistical analyses were performed using Stata version 16.0 (StataCorp). *P* values were reported as 2-tailed, and statistical significance was set at *P* < .05. Data were analyzed from June to August 2023.

## Results

Of the 125 188 participants included in the analysis, 1110 incident breast cancer cases were noted during a mean (range) follow-up of 6.7 (0.5-9.9) years (Figure 1). The mean (SD) age at enrollment was 34.8 (6.3) years (Table 1). The mean (SD) BMI was 21.6 (3.1; calculated as weight in kilograms divided by height in meters squared), and the mean (SD) waist circumference was 75.3 (8.2) cm. The proportion of first-degree relatives with a family history of breast cancer was 2.7% (3406 of 125 188). A total of 38 411 participants (30.7%) reported no history of childbirth, 31 205 (24.9%) had 1 child, 38 859 (31.0%) had 2 children, and 5106 (4.1%) had 3 children or more.

**Table 1.** Characteristics of the Study Population With Respect to Breast Cancer Development

Variables	Participants, No. (%)		
	Total (N = 125 188)	Breast cancer development	
		No (n = 124 078)	Yes (n = 1110)
Age at baseline, mean (SD), y	34.8 (6.3)	34.8 (6.3)	38.4 (5.1)
Follow-up time, mean (SD), y	6.7 (2.4)	6.7 (2.4)	4.9 (2.4)
Body mass index, mean (SD)^a^	21.6 (3.1)	21.6 (3.1)	21.5 (2.9)
Waist circumference, mean (SD), cm	75.3 (8.2)	75.3 (8.2)	75.7 (7.7)
Muscle mass, mean (SD), kg	37 (3.9)	37 (3.9)	37.2 (3.8)
Muscle mass/weight, mean (SD)	0.7 (0.6)	0.7 (0.1)	0.7 (0.1)
Fat mass, mean (SD), kg	16.7 (5.7)	16.8 (5.8)	16.3 (5.3)
Fat mass/weight, mean (SD)	0.3 (0.6)	0.3 (0.1)	0.3 (0.1)
Fat mass index, mean (SD)^b^	6.5 (2.3)	6.5 (2.3)	6.3 (2.1)
Family history of breast cancer			
No	121 752 (97.3)	120 700 (97.3)	1052 (94.8)
Yes	3406 (2.7)	3349 (2.7)	57 (5.1)
Missing	30 (0.0)	29 (0.0)	1 (0.1)
Age at menarche, y			
<16	102 400 (81.8)	101 521 (81.8)	879 (79.2)
16 or 17	11 032 (8.8)	10 952 (8.8)	80 (7.2)
>17	1413 (1.1)	1400 (1.1)	13 (1.2)
Missing	10 343 (8.3)	10 205 (8.2)	138 (12.4)
No. of children			
None	38 411 (30.7)	38 208 (30.8)	203 (18.3)
1	31 205 (24.9)	30 916 (24.9)	289 (26.0)
2	38 859 (31.0)	38 413 (30.9)	446 (40.2)
≥3	5106 (4.1)	5062 (4.1)	44 (3.9)
Missing	11 607 (9.3)	11 479 (9.2)	128 (11.5)
Patterns of physical activity (IPAQ short)			
Inactive	71 546 (57.2)	70 957 (57.2)	589 (53.1)
Minimally active	34 899 (27.9)	34 568 (28.9)	331 (29.8)
Health-enhancing physically active	16 356 (13.1)	16 194 (13.1)	162 (14.6)
Missing	2387 (1.9)	2359 (1.9)	28 (2.5)
Smoking status			
Never	99 364 (79.4)	98 577 (79.4)	787 (70.9)
Former	8545 (6.8)	8461 (6.8)	84 (7.6)
Current	3061 (2.4)	3031 (2.4)	30 (2.7)
Missing	14 218 (11.4)	14 009 (11.3)	209 (18.8)
Alcohol consumption			
No	17 957 (14.3)	17 782 (14.3)	175 (15.8)
1-2 Times/wk	86 520 (69.1)	85 790 (69.1)	730 (65.8)
≥3 Times/wk	9088 (7.3)	9021 (7.3)	67 (6.0)
Missing	11 623 (9.3)	11 485 (9.3)	138 (12.4)

Abbreviation: IPAQ, International Physical Activity Questionnaire.

^a^ Body mass index is calculated as weight in kilograms divided by height in meters squared.

^b^ Fat mass index is calculated as fat mass in kilograms divided by height in meters squared.

**Figure 2.**
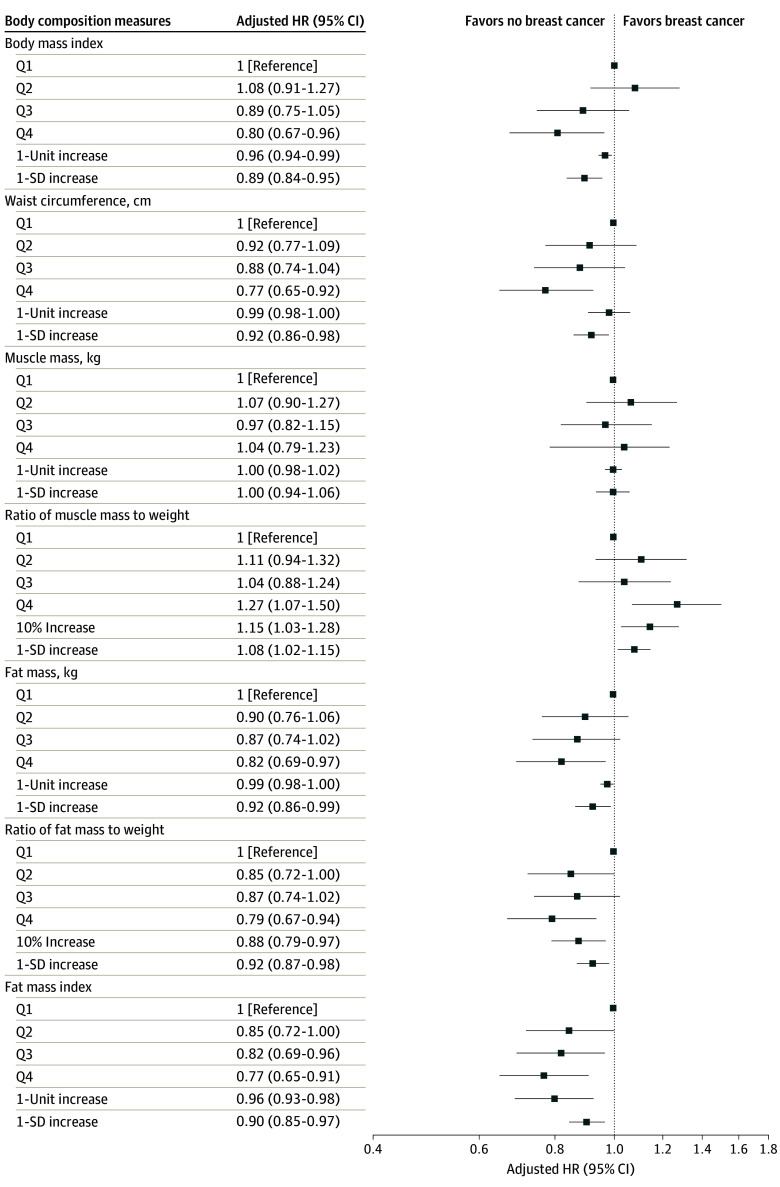
Association Between Body Mass Index, Body Composition Measures, and Premenopausal Breast Cancer Risk From the Multivariable Model HR indicates hazard ratio; Q, quartile.

A higher BMI was associated with decreased breast cancer risk, with an adjusted HR of 0.96 (95% CI, 0.94-0.99) per unit increase and 0.89 (95% CI, 0.84-0.95) per SD increase in BMI (Table 2 and Figure 2). Similar results were observed for waist circumference, with an adjusted HR of 0.99 (95% CI, 0.98-0.99) per unit increase and 0.92 (95% CI, 0.86-0.98) per SD increase; the adjusted HR for the highest quartile was 0.77 (95% CI, 0.65-0.92). No significant association was observed between muscle mass and the risk of premenopausal breast cancer. Conversely, increased breast cancer risk was positively associated with the ratio of muscle mass to weight in both models. In model 2, the adjusted HR (aHR) was 1.15 (95% CI, 1.03-1.28) per 10% increase and 1.08 (95% CI, 1.02-1.15) per SD increase. Women in the highest quartile had a 27% increase in breast cancer incidence compared with those in the lowest quartile (aHR, 1.27; 95% CI, 1.07-1.50). A consistent negative association with premenopausal breast cancer risk was observed when analyzing fat mass on the original scale, the ratio of fat mass to weight, and FMI. Specifically, women in the highest quartile compared with the lowest quartile had an aHR of 0.82 (95% CI, 0.69-0.97) for fat mass, 0.79 (95% CI, 0.67-0.94) for the ratio of fat mass to weight, and 0.77 (95% CI, 0.65-0.98) for FMI. Likewise, 1 SD increases in fat mass, ratio of fat mass to weight, and FMI were significantly associated with a reduced risk of breast cancer. Women in the highest quartile of fat mass had an 18% decrease in breast cancer risk, with an aHR of 0.82 (95% CI, 0.69-0.97). Breast cancer risk decreased by approximately 1.5% per kilogram increase in fat mass (aHR, 0.99; 95% CI, 0.98-0.99) and by 8% per SD increase in fat mass (aHR, 0.92; 95% CI, 0.86-0.99). Regarding the ratio of fat mass to weight, aHR for the highest quartile compared with the lowest quartile was 0.79 (95% CI, 0.67-0.94), and aHR per 10% increase was 0.88 (95% CI, 0.79-0.97). We observed a decrease in breast cancer as FMI increased, with aHRs in the third and fourth quartiles of 0.82 (95% CI, 0.69-0.96) and 0.77 (95% CI, 0.65-0.91), respectively, compared with the first quartile. The risk of breast cancer decreased by approximately 4% per unit increase in FMI (aHR, 0.96; 95% CI, 0.93-0.98) and by 10% per SD increase in FMI (aHR, 0.90; 95% CI, 0.85-0.97). The *P* for trend values of other body composition measures in model 2, except for muscle mass, were statistically significant (Table 2).

**Table 2.** Association of Body Composition Measures and Incident Breast Cancer in Premenopausal Women

Body composition measures	Person-years	Cases, No.	Incidence rate per 100 000 person-years (95% CI)	Breast cancer development, HR (95% CI)		
				Model 1^a^	Model 2^b^	Model 3^c^
Body mass index^d^						
Q1 (<19.5)	212 096	253	119 (105-135)	1 [Reference]	1 [Reference]	1 [Reference]
Q2 (19.5 to <21.3)	211 478	307	145 (130-162)	1.08 (0.91-1.28)	1.08 (0.91-1.27)	NA
Q3 (21.3 to >23.0)	211 976	283	134 (119-150)	0.88 (0.74-1.05)	0.89 (0.75-1.05)	NA
Q4 (≥23.0)	204 864	265	129 (115-146)	0.79 (0.67-0.95)	0.80 (0.67-0.96)	NA
C statistic (95% CI)	NA	NA	NA	0.67 (0.66-0.68)	NA	NA
Continuous per unit increase	NA	NA	NA	0.96 (0.94-0.99)	0.96 (0.94-0.99)	NA
Continuous per SD increase	NA	NA	NA	0.89 (0.83-0.95)	0.89 (0.84-0.95)	NA
* P* for trend^e^	NA	NA	NA	NA	.002	NA
Waist circumference, cm						
Q1 (<69.6)	199 828	247	124 (109-140)	1 [Reference]	1 [Reference]	1 [Reference]
Q2 (69.6 to <74.2)	209 219	276	132 (117-148)	0.92 (0.77-1.09)	0.92 (0.77-1.09)	0.89 (0.76-1.07)
Q3 (74.2 to <79.9)	214 863	303	141 (126-158)	0.88 (0.75-1.05)	0.88 (0.74-1.04)	0.85 (0.72-1.01)
Q4 (≥79.9)	211 104	279	132 (118-149)	0.77 (0.65-0.92)	0.77 (0.65-0.92)	0.74 (0.62-0.88)
C statistic (95% CI)	NA	NA	NA	0.67 (0.66-0.68)	NA	NA
Continuous per unit increase	NA	NA	NA	0.98 (0.98-0.99)	0.99 (0.98-0.99)	0.99 (0.98-0.99)
Continuous per SD increase	NA	NA	NA	0.92 (0.86-0.98)	0.92 (0.86-0.98)	0.90 (0.85-0.96)
* P* for trend^e^	NA	NA	NA	NA	.004	NA
Muscle mass, kg						
Q1 (<34.4)	213 555	259	121 (107-137)	1 [Reference]	1 [Reference]	1 [Reference]
Q2 (34.4 to <36.7)	202 915	279	137 (122-155)	1.07 (0.90-1.27)	1.07 (0.90-1.27)	0.98 (0.82-1.16)
Q3 (36.7 to <39.3)	211 240	273	129 (115-146)	0.97 (0.82-1.15)	0.97 (0.82-1.15)	0.83 (0.69-1.00)
Q4 (≥39.3)	209 489	296	141 (126-158)	1.03 (0.87-1.22)	1.04 (0.88-1.23)	0.82 (0.67-1.00)
C statistic (95% CI)	NA	NA	NA	0.67 (0.65-0.68)	NA	NA
Continuous per unit increase	NA	NA	NA	0.99 (0.98-1.01)	0.99 (0.98-1.02)	0.97 (0.95-0.99)
Continuous per SD increase	NA	NA	NA	0.99 (0.94-1.06)	0.99 (0.94-1.06)	0.89 (0.83-0.97)
* P* for trend^e^	NA	NA	NA	NA	.95	NA
Ratio of muscle mass (kg) to weight (kg)						
Q1 (<0.63)	197 744	246	124 (110-141)	1 [Reference]	1 [Reference]	1 [Reference]
Q2 (0.63 to <0.67)	208 643	284	136 (121-153)	1.11 (0.93-1.31)	1.11 (0.94-1.32)	1.09 (0.92-1.30)
Q3 (0.67 to <0.71)	212 281	261	123 (109-139)	1.04 (0.88-1.24)	1.04 (0.88-1.24)	1.02 (0.86-1.22)
Q4 (≥0.71)	218 531	316	145 (130-161)	1.26 (1.06-1.49)	1.27 (1.07-1.50)	1.23 (1.04-1.46)
C statistic (95% CI)	NA	NA	NA	0.67 (0.65-0.68)	NA	NA
Continuous per 10% increase	NA	NA	NA	1.14 (1.02-1.27)	1.15 (1.03-1.28)	1.12 (1.00-1.25)
Continuous per SD increase	NA	NA	NA	1.08 (1.01-1.14)	1.08 (1.02-1.15)	1.07 (1.00-1.14)
* P* for trend^e^	NA	NA	NA	NA	.01	NA
Fat mass, kg						
Q1 (<12.8)	221 858	304	137 (122-153)	1 [Reference]	1 [Reference]	1 [Reference]
Q2 (12.8 to <15.7)	209 640	273	130 (116-147)	0.90 (0.77-1.07)	0.89 (0.76-1.06)	0.89 (0.76-1.05)
Q3 (15.7 to <19.5)	209 618	278	133 (118-149)	0.87 (0.74-1.03)	0.87 (0.74-1.02)	0.86 (0.73-1.01)
Q4 (≥19.5)	196 083	252	129 (114-145)	0.82 (0.69-0.98)	0.82 (0.69-0.97)	0.80 (0.68-0.95)
C statistic (95% CI)	NA	NA	NA	0.67 (0.65-0.68)	NA	NA
Continuous per unit increase	NA	NA	NA	0.99 (0.98-0.99)	0.99 (0.98-0.99)	0.99 (0.97-0.99)
Continuous per SD increase	NA	NA	NA	0.92 (0.87-0.99)	0.92 (0.86-0.99)	0.91 (0.86-0.98)
* P* for trend^e^	NA	NA	NA	NA	.02	NA
Ratio of fat mass (kg) to weight (kg)						
Q1 (<0.25)	219 978	314	143 (128-159)	1 [Reference]	1 [Reference]	1 [Reference]
Q2 (0.25 to <0.29)	210 699	265	126 (112-142)	0.86 (0.73-1.01)	0.85 (0.72-0.99)	0.86 (0.73-1.01)
Q3 (0.29 to <0.33)	208 581	281	135 (120-151)	0.88 (0.75-1.03)	0.87 (0.74-1.02)	0.89 (0.76-1.05)
Q4 (≥0.33)	197 941	247	125 (110-141)	0.79 (0.67-0.94)	0.79 (0.67-0.94)	0.82 (0.69-0.97)
C statistic (95% CI)	NA	NA	NA	0.67 (0.65-0.68)	NA	NA
Continuous per 10% increase	NA	NA	NA	0.88 (0.79-0.98)	0.88 (0.79-0.97)	0.89 (0.81-0.99)
Continuous per SD increase	NA	NA	NA	0.93 (0.87-0.99)	0.92 (0.87-0.98)	0.94 (0.88-0.99)
* P* for trend^e^	NA	NA	NA	NA	.01	NA
Fat mass index^f^						
Q1 (<4.9)	216 010	303	140 (125-157)	1 [Reference]	1 [Reference]	1 [Reference]
Q2 (4.9 to <6.1)	211 822	269	127 (113-143)	0.85 (0.72-1.01)	0.85 (0.72-0.99)	NA
Q3 (6.1 to <7.5)	208 674	274	131 (117-148)	0.82 (0.69-0.97)	0.82 (0.69-0.96)	NA
Q4 (≥7.5)	200 684	261	130 (115-147)	0.77 (0.65-0.91)	0.77 (0.65-0.91)	NA
C statistic (95% CI)	NA	NA	NA	0.67 (0.65-0.68)	NA	NA
Continuous per unit increase	NA	NA	NA	0.96 (0.93-0.99)	0.96 (0.93-0.98)	NA
Continuous per SD increase	NA	NA	NA	0.91 (0.85-0.97)	0.90 (0.85-0.97)	NA
* P* for trend^e^	NA	NA	NA	NA	.002	NA

Abbreviations: NA, not applicable; Q, quartile.

^a^ Model 1 was adjusted for age at enrollment.

^b^ Model 2 was adjusted for age at enrollment, family history of breast cancer, age at menarche, parity, physical activity, smoking status, and alcohol consumption.

^c^ Model 3 was adjusted for variables similar to model 2, with additional adjustment for height (in centimeters, as a continuous variable).

^d^ Body mass index is calculated as weight in kilograms divided by height in meters squared.

^e^ *P* for trend was tested by fitting the body composition quartile variable as a single ordinal term in the model and was performed only for Model 2.

^f^ Fat mass index is calculated as fat mass in kilograms divided by height in meters squared.

Model 3 in Table 2 presents the HRs and 95% CIs for the associations with additional adjustments for height. The associations between waist circumference, the ratio of muscle mass to weight, fat mass, and fat mass to weight with breast cancer risk remained significant, with similar HR values when height was added to the regression model. However, after additionally adjusting for height, an increase in muscle mass, which was not associated with breast cancer risk, was associated with a decreased breast cancer risk, with an aHR of 0.97 (95% CI, 0.95-0.99) per unit increase and an aHR of 0.89 (95% CI, 0.83-0.97) per SD increase in muscle mass.

## Discussion

In this prospective study of premenopausal women, a relatively higher body fat mass, fat mass-to-weight ratio, and FMI were associated with a decreased risk of breast cancer. There was no association between muscle mass and breast cancer risk; however, a positive association was observed between the ratio of muscle mass to weight and an elevated risk of breast cancer. Specifically, we found an 8% decrease in breast cancer risk per SD increase in fat mass and an 8% decrease per SD increase in the ratio of fat mass to body weight. However, an SD increase in the ratio of muscle mass to weight was associated with a 1% increase in breast cancer risk, and a 10% increase in the ratio of muscle mass to weight was associated with an approximately 15% increase in breast cancer risk. Greater associations were observed between FMI and decreased breast cancer risk. These associations remained significant even after adjusting for height.

The strength of this study was its large sample size of premenopausal Korean women. The prospective cohort design enabled us to measure exposure before breast cancer development, thus minimizing reverse causality or selection bias. Ascertainment of the incidence of breast cancer cases was highly accurate because it was obtained by linkage with national cancer statistics, and cases were recorded independent of participation in this study. Our study also benefited from the high-quality assessment of anthropometric and body composition measurements, which were performed by trained nurses or laboratory technicians instead of using self-reported information.

In this study, we first evaluated the association between conventional adiposity measures, including BMI and waist circumference, and breast cancer risk. Our results are consistent with the findings of previous studies^27,28^ and a large pooled analysis^3^ showing decreased breast cancer risk in premenopausal women with a higher BMI or higher waist circumference. The large pooled analysis study further found stronger inverse associations of risk with BMI in younger than in older persons (aHR per 5-unit increase in BMI for ages 18-24 years was 0.77; 95% CI, 0.73-0.80 vs aHR, 0.88; 95% CI, 0.86-0.91 for ages 45-54 years).^3^ Meanwhile, no significant inverse association between BMI and premenopausal breast cancer risk was found in other studies,^11,29,30^ highlighting the inconsistent findings regarding the relationship between adiposity and breast cancer in this population. Heterogeneity in the association between obesity and breast cancer risk may be influenced by various factors, including race or hormone receptor status. In a pooled analysis involving African American and White women, the BMI at 18 years of age was inversely associated with the risk of premenopausal breast cancer. Importantly, this inverse association remained consistent across estrogen receptor, progesterone receptor, and ERBB2-defined subtypes and did not vary by race (White or African American women).^31^ Conversely, in another cross-sectional study focusing exclusively on African American premenopausal women, a positive association was observed between adiposity, assessed using the waist-to-hip ratio, and young-onset breast cancer.^32^ The study highlighted that African American individuals with early onset breast cancer tend to exhibit a higher frequency of poor prognosis tumor characteristics, including negative estrogen and progesterone receptor status, as well as the triple-negative subtype. Due to the unavailability of clinical characteristics, such as hormone receptor information, this study did not account for these factors. Future studies investigating the association between body composition measures and different clinical characteristics of premenopausal patients with breast cancer are warranted. Notably, our cohort was relatively younger than those in previous studies on this topic, where no significant associations were found; the mean age at baseline in our study was 35 years, whereas it was 45 years in the European Prospective Investigation into Cancer and Nutrition study^29^ and 44 years in the Women’s Circle of Health Study.^30^ This age difference may explain the discrepant findings, as a stronger association between increased BMI and decreased breast cancer risk in younger women^3^ has been reported.

As BMI has limitations on assessing breast cancer risk because it does not reflect the body fat distribution and overall body fat level, recent studies^10,11,33^ have begun assessing breast cancer risk associated with other body shape and body fat distribution indicators. While higher body fat mass was associated with breast cancer risk in postmenopausal women,^10,11,33^ the association remains unclear in premenopausal women. Before our study, some studies among Western populations reported an overall negative association between larger body shape during adulthood and breast cancer in young women,^28,34,35^ while another study suggested that body fat does not play an important role in premenopausal breast cancer.^36^ However, evidence from these studies was based on conventional measurements of body shape, including waist circumference, hip circumference, and waist-to-hip ratio; studies on body fat mass remain limited. Only 1 recent study using data from the UK Biobank assessed the association between whole-body fat mass assessed using bioelectrical impedance and fat mass in different parts of the body and breast cancer risk in premenopausal women; however, no significant association was found.^11^ Again, our cohort was relatively younger than the premenopausal population from the UK Biobank cohort (35 vs 46 years), which may explain the different findings between the 2 studies in terms of BMI and breast cancer risk. Additionally, the younger age of our cohort was a unique characteristic that distinguished our findings from those of previous studies on this topic.

Fat-free mass, which comprises the muscles, is another major component of body composition, and recent interest has centered on the importance of muscle mass for normal physiological function.^37^ We found no association between muscle mass and premenopausal breast cancer; however, increased breast cancer risk was associated with a higher muscle mass–to-weight ratio. Several mechanisms may account for the discrepancy in the results between the models of muscle mass alone and muscle mass over weight. First, the difference in the results between the models of muscle mass alone and the ratio of muscle mass to weight could be attributed to the confounding variable of fat or overall body weight. Individuals with higher body weight generally exhibit greater muscle mass. In a model focusing solely on muscle mass, the association with breast cancer may be confounded by the presence of fat or overall body weight. Conversely, in the model emphasizing the ratio of muscle mass to weight, the outcome of weight was somewhat controlled. When additionally adjusted for height (model 3), the different associations between muscle mass, muscle mass-to-weight ratio, and breast cancer risk were more evident, suggesting confounding outcomes of body fat or weight. A recent study identified that the skeletal muscle index, which was defined as skeletal muscle mass divided by weight, was more associated with breast density than BMI,^38^ with a strong association between skeletal muscle and breast parenchymal tissue. The association between increased fat mass, BMI, and decreased breast density,^39^ as well as the association between increased muscle mass and increased breast density,^38^ could partially explain the association between body composition and breast cancer risk in premenopausal women. Analysis of the Karolinska Mammography Project for Risk Prediction of Breast Cancer cohort^40^ suggested that hormones from the progestogen, estrogen, and corticoid pathways are associated with breast density and that changes in breast density over time are mainly driven by androgens, which possess potent anabolic effects on skeletal muscle. Although this study did not consider mammographic density in the association between body composition and breast cancer risk, further studies are required to elucidate the biological link between body composition and breast cancer risk.

Thus far, it has been suggested that breast cancer due to obesity in premenopausal women may have different biological mechanisms than that in postmenopausal women. One possible explanation for the different associations between obesity and breast cancer risk by menopausal status could be the higher estrogen levels in postmenopausal obese women. In premenopausal obese women, estrogen synthesis from fat accounts for a small proportion of total estrogen, while estradiol from fat accounts for an increased proportion in postmenopausal women in whom estrogen from ovarian function is absent.^41,42^ Another study further suggested that estrogen from fat, together with estrogen from the ovary, results in negative feedback on the hypothalamic-pituitary axis. Decreased levels of progesterone, which is involved in breast tissue proliferation due to hypothalamic-pituitary suppression, are responsible for the reduced breast cancer risk in obese premenopausal women.^43^ Additional suggested mechanisms include a synergistic interactive effect between breast density and BMI in premenopausal women^42,44,45^ and varying roles of leptin, with elevated levels increasing breast cancer risk in postmenopausal women but not affecting or decreasing the risk in premenopausal women.^42,46,47^ Another mechanism might be the different role of insulin resistance, showing a positive association between type 2 diabetes and breast cancer risk in postmenopausal women, independent of BMI, but not in premenopausal women.^42,48^

It is worth noting that the age distribution of breast cancer risk in the Korean population differs from that in the Western population. In Korean women, the breast cancer risk incidence rate peaked in the age group of 40 to 49 years and decreased as age increased,^49^ which is contrary to findings in the Western population, where increasing age is associated with an increased risk of breast cancer.^50^ Therefore, evidence of the factors associated with premenopausal breast cancer risk is important in Asian women, where premenopausal breast cancer accounts for a higher proportion of breast cancers.

### Limitations

This study has some limitations. First, our study participants were selected from a health screening environment, which potentially restricts the applicability of our results to the entire Korean female population. Second, the impedance-based body composition test, although noninvasive, has limitations, potentially overestimating fat-free mass and underestimating fat mass in obese elderly populations.^17^ Additionally, BIA measurements can be influenced by factors such as fluid status, pregnancy, and malnutrition.^51^ Although we did not assess the participants’ hydration status before the body composition assessment, all participants observed an overnight fast of 10 or more hours before BIA measurements to align with fasting blood sample collection. However, any potential inaccuracies in the BIA assessment applied uniformly to all study participants. Third, owing to the observational nature of our study, we could not establish a cause-and-effect link between body composition measures and the risk of breast cancer in premenopausal women. Third, unmeasured factors that were not considered in our analysis may have influenced our findings. Additionally, our datasets lacked information on breast cancer molecular subtypes and stages, which limited our ability to further explore these aspects.

## Conclusions

In this cohort study of Korean women, a negative association was observed between adiposity, fat mass, and the risk of developing premenopausal breast cancer. Further research is necessary to gain a deeper understanding of the underlying mechanisms of this association and to characterize the influence of body fat distribution on premenopausal breast cancer risk in other Asian populations.

## Notes

Supplement.
Data Sharing Statement
