# Supplementary material for: Adiposity, Body Composition Measures, and Breast Cancer Risk in Korean Premenopausal Women
Source: JAMA Netw Open. 2024 Apr 5;7(4):e245423. doi: 10.1001/jamanetworkopen.2024.5423 (PMC10998159; doi:10.1001/jamanetworkopen.2024.5423)
Supplement: Supplement. — Data Sharing Statement [file jamanetwopen-e245423-s001.pdf]

Data Sharing Statement

Tran. Adiposity, Body Composition Measures, and Breast Cancer Risk in Korean Premenopausal Women. *JAMA Netw Open*. Published April 05, 2024. doi:10.1001/jamanetworkopen.2024.5423

Data
Data available: No

Additional Information
Explanation for why data not available: Data were obtained with a data-use agreement from the Kangbuk Samsung Health Study Cohort. Therefore, we are unable to make the data publicly available.
